# Effect of Mirror Therapy on Post-Needling Pain Following Deep Dry Needling of Myofascial Trigger Point in Lateral Elbow Pain: Prospective Controlled Pilot Trial

**DOI:** 10.3390/jcm13051490

**Published:** 2024-03-05

**Authors:** Sebastián Eustaquio Martín Pérez, Jhoselyn Delgado Rodríguez, Alejandro Kalitovics, Pablo de Miguel Rodríguez, Daniela Sabrina Bortolussi Cegarra, Iremar Rodríguez Villanueva, Álvaro García Molina, Iván Ruiz Rodríguez, Juan Montaño Ocaña, Isidro Miguel Martín Pérez, María Dolores Sosa Reina, Jorge Hugo Villafañe, José Luis Alonso Pérez

**Affiliations:** 1 Musculoskeletal Pain and Motor Control Research Group, Faculty of Health Sciences, Universidad Europea de Canarias, 38300 Santa Cruz de Tenerife, Spain; jhoselin_7@hotmail.com (J.D.R.); alejandrokd15@gmail.com (A.K.); pablodmr.fisio@gmail.com (P.d.M.R.); joseluis.alonso@universidadeuropea.es (J.L.A.P.); 2 Musculoskeletal Pain and Motor Control Research Group, Faculty of Sport Sciences, Universidad Europea de Madrid, Villaviciosa de Odón, 28670 Madrid, Spain; danielabortolussi@hotmail.com (D.S.B.C.); iremar.rv@gmail.com (I.R.V.); agarciamolina19@gmail.com (Á.G.M.); ivan.ruiz.rodriguez@gmail.com (I.R.R.); i.montano.ocana@facultyue.es (J.M.O.); mariadolores.sosa@universidadeuropea.es (M.D.S.R.); mail@villafane.it (J.H.V.); 3 Departamento de Medicina Física y Farmacología, Área de Radiología y Medicina Física, Facultad de Ciencias de la Salud, Universidad de la Laguna, 38200 Santa Cruz de Tenerife, Spain; 4 Escuela de Doctorado y Estudios de Posgrado, Universidad de La Laguna, 38200 Santa Cruz de Tenerife, Spain; 5 Department of Physiotherapy, Faculty of Sport Sciences, Universidad Europea de Madrid, Villaviciosa de Odón, 28670 Madrid, Spain

**Keywords:** mirror therapy, dry needling, post-needling pain, myofascial trigger point

## Abstract

**Background**: This prospective randomized, controlled pilot trial to explore the immediate effect of adding Mirror Visual Feedback Therapy on pain sensitivity and motor performance among subjects suffering from post-needling pain diagnosed as Lateral Elbow Pain. **Methods**: A total of 49 participants (23 female, 26 male) were enrolled and randomly allocated to either the experimental group, which received Deep Dry Needling in the m. *Brachioradialis*, Ischemic Compression, Cold Spray, Stretching, and Mirror Visual Feedback Therapy (*n* = 25), or a control group without Mirror Visual Feedback Therapy (*n* = 24). Pre- and post-treatment evaluations included assessments of post-needling pain intensity, pressure pain threshold, two-point discrimination threshold, and maximum hand grip strength. **Results**: Intergroup analysis revealed a statistically significant reduction in post-needling pain intensity favoring the experimental group (U = 188.00, *p* = 0.034). Additionally, intragroup analysis showed significant improvements in post-needling pain intensity (MD = 0.400, SEM = 0.271, W = 137.00, *p* = 0.047) and pressure pain threshold (MD = 0.148 Kg/cm^2^, SEM = 0.038, W = 262.00, *p* < 0.001) within the experimental group following the intervention. **Conclusions**: These findings suggest a potential benefit of integrating Mirror Visual Feedback Therapy into treatment protocols for individuals with Lateral Elbow Pain experiencing post-needling discomfort. Further research is necessary to fully elucidate the clinical implications of these findings.

## 1. Introduction

Lateral Elbow Pain, affecting 1–3% of the population annually, is a common musculoskeletal disorder primarily associated with occupational activities and repetitive biomechanical stress on the joints, including the intricate support systems within the hand and wrist [1,2]. Myofascial pain syndrome (MPS) emerges as a significant etiological factor in Lateral Elbow Pain, characterized by the presence of hyperirritable and taut bands within extensor muscles, known as myofascial trigger points (MTrPs), which can lead to local pain and regional sensory abnormalities [3,4].

These symptoms may arise from the activation of sensory–motor pathways within the spinal cord and brainstem by MTrPs [5], leading to the involvement of reflex circuits that induce local spastic responses, referred pain, and motor dysfunction in individuals diagnosed with MPS [6,7,8,9,10]. Additionally, there is a suggested association between sensory abnormalities and peripheral sensitization triggered by active muscle nociceptors [11,12,13]. Consequently, the persistent activation of these nociceptors is thought to contribute to the progression of central sensitization (CS) [14]. Although the exact mechanism remains elusive, recent studies have implicated ionotropic acid-sensing ion channels (ASIC), specifically ASIC-1 and ASIC-3, in the development of CS [15]. These channels play distinct roles in primary and secondary hyperalgesia, indicating their potential involvement in facilitating the transition from peripheral to central sensitization [16].

Conservative approaches such as Deep Dry Needling (DDN) have demonstrated effectiveness in reducing hyperalgesia and alleviating sensory–motor disturbances [17,18]. This technique involves rhythmic transcutaneous needling of the affected muscles or MTrPs without the use of injectable substances [19]. Recent studies examining biopsies from animal models post Deep Dry Needling (DDN) have shown an increase in the release of neurotransmitters and proinflammatory substances in the punctured area. These include endogenous opioids, which inhibit pain transmission [20], and a reduction in substances such as bradykinin, substance P, CGRP, TNF, IL-1β, serotonin, and norepinephrine [21,22,23]. Additionally, pH increases in the MTrPs region deactivate acid-sensing ion channels, potentially reducing mechanical hyperalgesia and limiting CS onset [24].

Nevertheless, in human models, it is commonly observed that pain arises as an iatrogenic complication subsequent to puncture procedures, often referred to as post-needling pain. Its incidence is typically estimated to range from 29.4% to 100% in most studies and it usually arises immediately following the procedure and diminishes within 72 h [25,26,27], a timeframe that can be attributed to the immediate release of algogenic substances triggering an inflammatory response and neuromuscular damage caused by the needle [28]. This primary peripheral tissue sensitization that arises from dysfunctional sensory signals at the puncture site, compounded by spinal dysregulation, could cause heightened pain signal transmission to the central nervous system (CNS). Moreover, spinal dysregulation can result in heightened nociceptor activity, deterioration of C-fibers, depletion of A-fibers, adverse modulation of GABAergic activity, and opioid receptors, expanded receptive fields in deafferented areas, and spinal cord hyperexcitability due to modified substance P expression by Aß fibers [29,30]. This may elucidate the pronociceptive effect experienced by patients following treatment with DDN.

For its management, the gold standard involves maneuvers of Ischemic Compression (IC) combined with Cold Spray with Stretching (STR) because of their efficacy in controlling blood perfusion, oxygen and nutrient supply, and activation of endogenous analgesic mechanisms [31,32]. However, the analgesic therapeutic effects achieved through the aforementioned procedure could be further augmented by stimulating supramedullary centers responsible for processing observational and movement execution. In this context, Mirror Visual Feedback Therapy (MVF) emerges as an effective, safer, and cost-efficient technique for activating brain areas associated with the mesolimbic system and the somatosensory cortex [33,34]. These areas play a crucial role in generating perceptual illusions [35] and recalibrating sensory–motor integration [36], potentially leading to a reduction in nociceptive pain and sensory–motor functional impairment after DDN [37,38,39].

While MVF has demonstrated promise in mitigating pain and reinstating voluntary motor functions among patients diagnosed with stroke [40] or phantom limb syndrome [41,42], its immediate effects in managing post-needling pain have not yet been explored in the literature. Due to the lack of comparable research examining and elucidating the immediate physiological impact of integrating MVF with the gold standard treatment following DDN, this pilot study aims to explore the immediate effect of adding MVF on pain sensitivity and motor performance among subjects suffering from post-needling pain diagnosed as Lateral Elbow Pain.

## 2. Materials and Methods

### 2.1. Study Design

**Figure 1:**
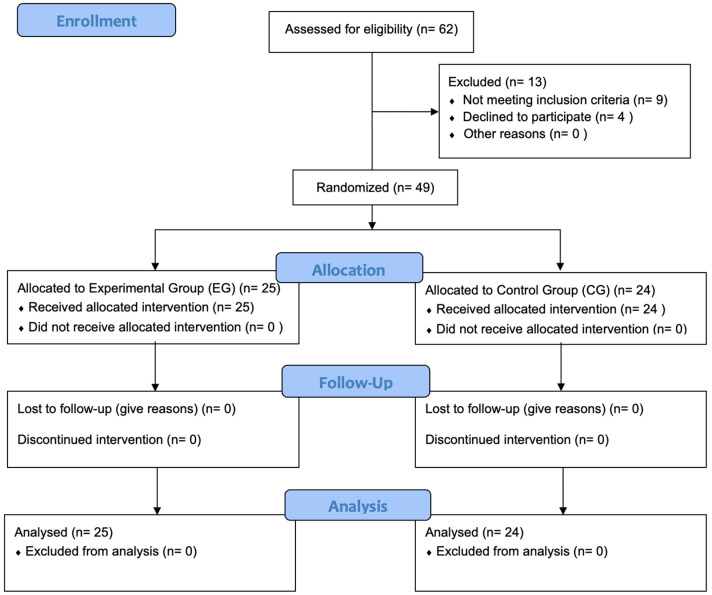
CONSORT flow diagram. Flowchart showing enrollment, allocation, follow-up and analysis throughout the study.

A prospective randomized, controlled, two-arm pilot trial was conducted between 1 February 2023 and 2 June 2023, rigorously adhering to the Consolidated Standards of Reporting Trials standard (CONSORT) [43] (Table S1). The study was conducted in compliance with the principles outlined in the Declaration of Helsinki and received approval from the Ethics Committee of Hospital Clínico San Carlos in Madrid, Spain (approval number 23/107-EC X TFM, dated 7 March 2023). Additionally, the study was prospectively registered on ClinicalTrials.gov with the identifier NCT06288048. The study protocol was developed by researchers D.S.B.C., I.R.V., and A.G.M.; A.K., J.D.R., and P.d.M.R. were responsible for administering written informed consent, intervention and assessments. Participant selection is described below in Figure 1, in a CONSORT Flow Diagram. 

### 2.2. Participants

Participants were recruited through consecutive non-probabilistic sampling from 20 February 2023 to 1 June 2023. The recruitment strategy employed a multifaceted approach, which included word-of-mouth referrals from both physical therapy and general practitioner (GP) consultations, outreach through social media platforms, posting notices on bulletin boards, and leveraging existing researcher networks. The inclusion criteria were established as follows:

(1) Males and females aged 18 years or older, (2) suffering from lateral elbow myofascial pain diagnosed by either a GP or physical therapist (3) for a duration of less than 3 months, (4) lacking a history of severe trauma, (5) having no prior exposure to Dry Needling treatment, (6) not currently using relevant medications, (7) having no history of musculoskeletal surgeries and (8) no documented toxic habits.

All participants provided written consent prior to participating in the study.

### 2.3. Sample Size Determination

The G*Power 3.1 software was utilized for the computation of sample size and power [44]. The estimations were based on a minimal clinically important difference (MCID) in the Visual Analog Scale (VAS) of 30 mm established for healthy subjects [45]. Assuming a confidence interval (CI) of −36.4 to −23.6, a two-tailed test, an alpha level of 0.05, and a desired power of 80%, the estimated sample size for each arm was 23 individuals.

### 2.4. Randomized Allocation

Participants were randomly allocated to either the intervention or control groups using a number sequence generated by an independent researcher. The randomization process was conducted using a random sequence generator available at Random.org (http://www.random.org (accessed on 1 April 2023). Allocation concealment was appropriately maintained throughout the study.

### 2.5. Intervention

The intervention was conducted in the simulated hospital consultation room at the European University (Madrid, Spain) during a single 1 h long session. Prior to the intervention, the practitioner responsible for administering the treatment, possessing over 10 years of clinical experience, conducted an interview to validate the inclusion criteria. Furthermore, clinical assessment maneuvers, including flat and pincer palpation, were carried out on the elbow extensor muscles to identify regions of augmented mechanical hyperalgesia corresponding to m. *Brachioradialis* (BR) MTrP patient symptoms. Afterwards, when obtaining informed consent, clear and consistent instructions regarding treatment efficacy were provided to both groups. Subsequently, participants were randomly allocated according to blinded sequence into two distinct treatment groups.

Experimental group: Deep Dry Needling (DDN), Ischemic Compression, Cold Spray with Stretching + MVF.

The DDN intervention targeted the proximal third of the m. *Brachioradialis* (BR) with the patient seated, and the therapist positioned on the same side as the needle insertion. Dry Needling involved a lateral-to-medial needle insertion direction toward the clinician’s finger, with a precision grip. In lean patients, precautions were taken to avoid accidental finger puncture by inserting the needle between the clinician’s fingers. DDN was performed with needle (AguPunt^®^, Barcelona, Spain) seeking three local twitch responses in m. *Brachioradialis* (BR). For subjects without such responses, 10 needle insertions and withdrawals at a frequency of 1 Hz were performed.

Following needling, Ischemic Compression (IC) was applied using a sphygmomanometer on the seated subject’s arm. Pressure was increased until ischemic pain appeared (approximately 200 mmHg), maintained for 90 s. This was combined with three applications of Cold Spray (Cryos Phyto Performance 400 mL) from origin to insertion, synchronized with m. *Brachioradialis* (BR) stretching consisting of passive sustained mobilization with elbow extension and forearm pronation for 10 s.

The intervention concluded with Mirror Visual Feedback Therapy (MVF). The patient was seated with forearms resting on the bed, facing a 35 × 35 cm mirror (Mirror Therapy Box, Reflex Pain Management Ltd®, Stockport, UK) covering the punctured side at a 45-degree angle for proper hand visualization. The punctured limb was positioned behind the mirror, out of the subject’s view. Any identifying objects (rings, bracelets, etc.) on the healthy limb were removed or covered. The MVF protocol consisted of two phases, each lasting 20 min. In the first phase, the therapist performed hand exercises while the subject observed the therapist’s hand reflected in the mirror. In the second phase, the subject executed hand opening and closing and wrist extension and flexion movements while observing their hand in the mirror. All movements were conducted at a frequency of 1 Hz with 40 repetitions.

Control group: Deep Dry Needling (DDN), Ischemic Compression, Cold Spray with Stretching.

**Figure 2:**
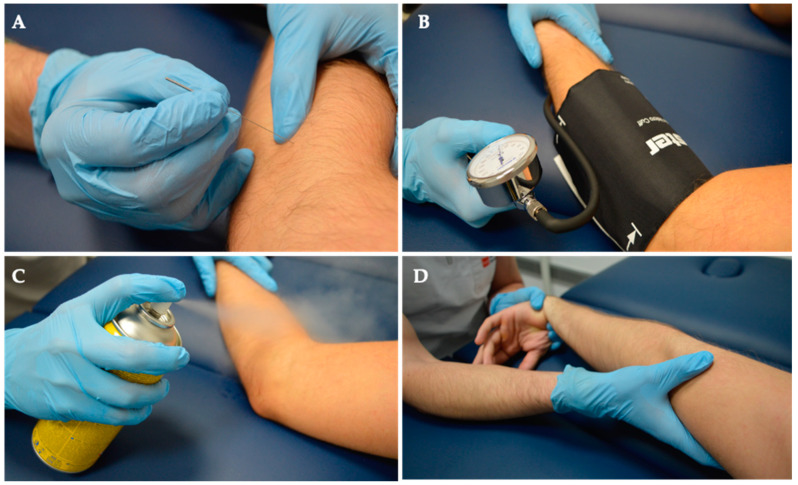
Intervention. Both groups received a comprehensive treatment regimen consisting of (**A**) Deep Dry Needling, (**B**) Ischemic Compression, (**C**) Cold Spray application, and (**D**) Stretching exercises.

**Figure 3:**
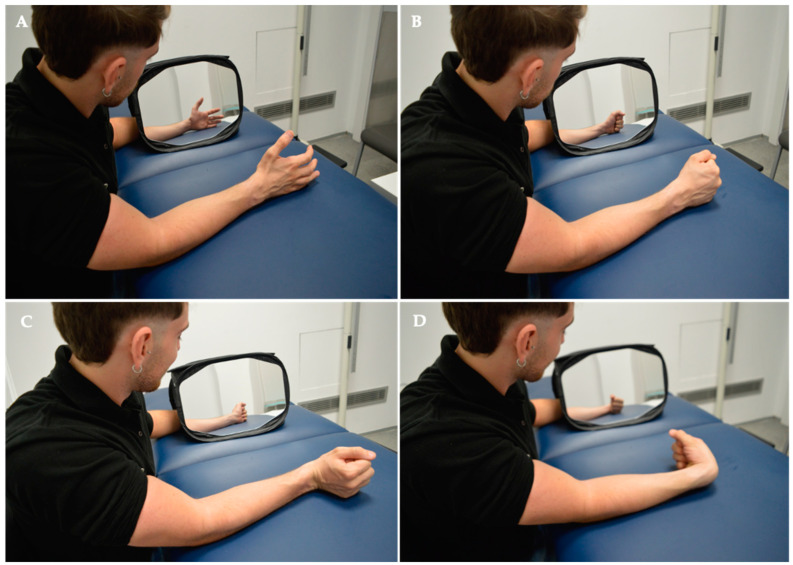
Intervention. Mirror Visual Feedback Therapy programme implemented for the experimental group. In the MVF Observation Phase for MTrPS Lateral Elbow Pain, specific muscle functions were targeted, including (**A**) hand opening, (**B**) hand closing, (**C**) wrist extension, and (**D**) wrist flexion.

Group 2 underwent Deep Dry Needling (DDN), Ischemic Compression, Cold Spray, and Stretching. Notably, this group did not undergo MVF. See details below, in Figure 2 and Figure 3.

### 2.6. Outcome Measures

The assessments encompassed pain intensity (VAS), pain pressure threshold (Wagner™ FPX Algometer 50, Greenwich, CT, USA), two-point discrimination threshold (Baseline^®^ 12-1480 skin caliper, 2-point discriminator, White Plains, NY, USA), and maximum hand grip strength (JAMAR^®^ Hand Dynamometer J00105, Bolingbrook, IL, USA). Measurements were documented both before starting the intervention and within 5 min after concluding the treatment for pre- and post-intervention assessments. All outcomes were evaluated by an assessor blinded to the treatment allocation of the subjects.

### 2.7. Statistical Analysis

Descriptive statistics were computed, encompassing measures of central tendency and dispersion parameters. To assess the normality of the data, the Shapiro–Wilk test was systematically applied to all study variables. Following the verification of data normality, intra-group variations were quantified utilizing the Wilcoxon signed-rank test, while inter-group differences were appraised through the Mann–Whitney U test. Subsequently, the calculation of effect size was performed using the point–biserial correlation coefficient. All statistical analyses were executed with the SPSS 26.0 software (IBM Corp.^®^, Armonk, NY, USA). Significance was established considering *p*-values below 0.05 as indicative of statistical significance.

## 3. Results

### 3.1. Demographic Description of the Sample

A total of 49 participants (F = 23, M = 26) were selected, with an average age of 24.9 (10.9) in the EG (n = 25) and 24.9 (9.5) years in the CG (n = 24). The mean weight of participants was 75.8 (5.7) Kg for the EG and 73.3 (10.5) Kg for the CG. Regarding height, the EG had a slightly higher stature at 1.77 (0.06) m compared to the CG at 1.72 (0.08) m. The body mass index (BMI) was very similar between the two groups, with values of 24.2 (1.7) and 24.7 (3.5), respectively. No statistically significant differences were identified between the two experiment groups. Further details can be found in Table 1.

### 3.2. Description of Study Variables

In terms of pre- and post-needling pain intensity (VAS 0-10), the experimental group showed a mean of 1.416 and 1.064, respectively, with the control group showing values of 1.150 and 1.633. PPT (Kg/cm^2^) indicated mean values of 1.485 Kg/cm^2^ (pre) and 1.77 Kg/cm^2^ (post) for the experimental group, and 1.843 Kg/cm^2^ (pre) and 1.72 Kg/cm^2^ (post) for the control group. Additionally, Two-point discrimination thresholds (TPDT) in millimeters revealed pre-values of 12.3 mm (EG) and 12.7 mm (CG), with post-values of 13.5 mm and 13.7 mm, respectively. Maximum hand grip strength (MHGS) displayed pre-values of 37.96 Kg/F for EG and 34.04 Kg/F in CG, while post-values were 37.712 Kg/F and 34.867 Kg/F. Due to the presence of violations of normality assumptions, a decision was made to employ non-parametric analysis for examining intra-group and inter-group differences in the study. More details are in Table 2.

### 3.3. Main Findings

#### 3.3.1. Intra-Group Differences

Experimental group: Deep Dry Needling (DDN), Ischemic Compression, Cold Spray with Stretching + MVF.

Post-needling pain intensity in participants of the experimental group (EG) showed statistically significant findings (MD = 0.400, SEM = 0.271, W = 137.00, *p* = 0.047) immediately after the addition of MVF to the DDN protocol. Similarly, the pressure pain threshold (PPT) demonstrated significant results (MD = 0.148 Kg/cm^2^, SEM = 0.271, W = 262.00, *p* < 0.001). In contrast, the two-point discrimination threshold (TPDT) (MD = −0.000 mm, SEM = 0.674, W = 121.00, *p* = 0.711) and maximum hand grip strength (MHGS) (MD = 0.200 Kg/F, SEM = 0.1999, W = 177.00, *p* = 0.224) did not exhibit statistically significant differences between initial and post-treatment measurements. Intra-group differences of the experimental group are detailed in Table 3.

Control group: Deep Dry Needling (DDN), Ischemic Compression, Cold Spray with Stretching.

Post-needling pain intensity in participants assigned to the control group (CG), as assessed by the Visual Analog Scale (VAS 0-10), did not reveal statistically significant differences (MD = −0.400, SEM = 0.247, W = 105.50, *p* = 0.643). Similarly, the pressure pain threshold (PPT) displayed non-significant results (MD = 0.029 Kg/cm^2^, SEM = 0.073, W = 163.00, *p* = 0.648), as did the two-point discrimination threshold (TPDT) (MD = 1.999 mm, SEM = 1.047, W = 207.00, *p* = 0.983) following 5 min of control intervention. Conversely, in the case of maximum hand grip strength (MHGS), there was a notable trend toward improvement in the CG, although without reaching statistical significance (MD = −0.799 Kg/F, SEM = 1.051, W = 83.00, *p* = 0.081). Intra-group differences of control group are displayed in detail in Table 4.

#### 3.3.2. Inter-Group Differences

**Figure 4:**
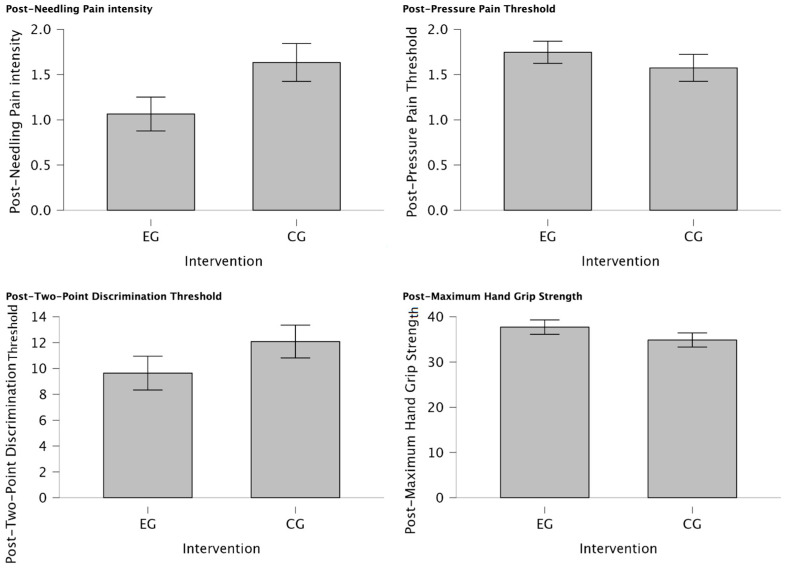

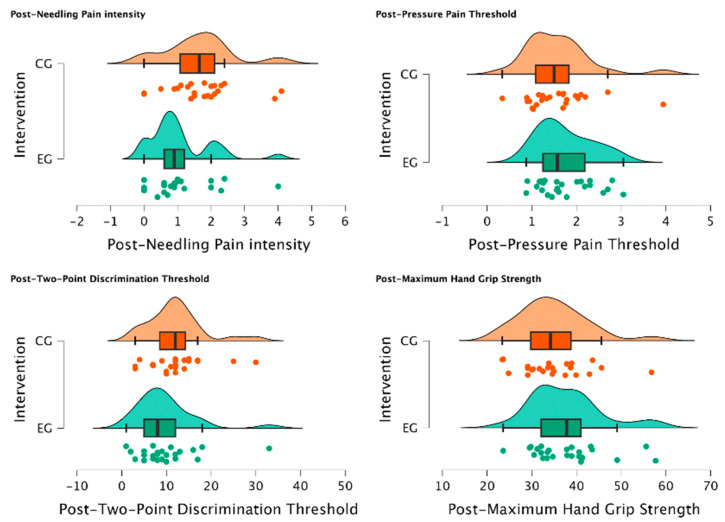
Intergroup differences between EG vs. CG after intervention. Significant reduction in post-needling pain intensity (VAS 0-10) (*p* = 0.034) was observed in individuals treated with MVF compared to those who were not, indicating a shift towards lower pain levels. Additionally, a notable group difference was observed in post-TPDT (mm) (*p* = 0.079) values, with substantial reduction in values among EG participants. However, post-PPT (Kg/cm^2^) and post-MHGS (Kg/F) values showed non-significant differences between groups (*p* = 0.862, *p* = 0.905, respectively).

There was a statistically significant reduction in post-needling pain intensity (VAS 0-10) (U = 188.00, *p* = 0.034) suggesting a meaningful shift towards lower pain levels in individuals of EG treated with MVF with those who were not (CG) for managing post-needling pain. Furthermore, post-TPDT (mm) exhibited a trend towards significance (U = 212.00, *p* = 0.079), signifying a notable group difference and a substantial reduction in values among participants assigned to the EG. In contrast, post-PPT (Kg/cm^2^) and post-MHGS (Kg/F) values showed non-significant distinctions between groups (U = 354.00, *p* = 0.862; U = 365.0, *p* = 0.905, respectively). Detailed explanations of intergroup differences are provided in Table 5 and visually depicted in Figure 4.

## 4. Discussion

The addition of Mirror Visual Feedback Therapy (MVF) after Dry Needling (DDN) in patients experiencing post-needling pain from myofascial trigger points (MTrPs) in Lateral Elbow Pain has shown promise in reducing pain and enhancing sensitivity in patients with Lateral Elbow Pain. The study’s findings revealing a statistically significant reduction in post-needling pain intensity within the experimental group (EG) receiving MVF compared to the control group (CG) not only underscores the potential efficacy of MVF, but also suggests its utility in managing acute pain associated with DDN in MTrP patients.

This convergence of results with prior research emphasizes the role of visual feedback mechanisms in pain modulation and the improvement of sensory function across a spectrum of musculoskeletal disorders [46,47]. Indeed, current theories support the notion that visualizing symmetrical movements through a mirror may influence sensory perception and modulate neuronal activity associated with pain sensation [48].

Firstly, this could be explained due to MVF showing the activation of mirror neuron systems which facilitate ipsilateral primary motor cortex (M1) excitability [49,50]. Furthermore, increased corticospinal excitability of the M1 has been observed to be associated with more efficient inhibitory pain modulation assessed by CPM [51]. In MPS, such as in our patients, M1 excitability exhibits characteristics such as reduced cortical silent period, decreased short-interval intracortical inhibition, and heightened intracortical facilitation and motor-evoked potentials [52]. Based on our findings, MVF appears to hold promise in diminishing corticospinal excitability, which encompasses resting motor threshold, MEPs, and cortical silent period [53].

Secondly, an alternative hypothesis explaining the immediate relief from pain and increasing mechanical threshold involves the influence of Distraction and Attentional Modulation [54,55,56,57]. The visual feedback provided by MVF can act as a distracting element, diverting the patient’s attention away from the sensation of pain [33]. Recent research suggests that the perception of the illusion triggers activation in the motor cortex by providing strong visual feedback that movements are occurring naturally and without hindrance. Moreover, this visual illusion enhances activity in the primary motor cortex (M1) through a top–down effect on the ipsilateral premotor cortex, thereby facilitating increased neuroplasticity [58]. Additionally, mirror-induced visual illusions stimulate various brain regions including the fronto-temporo-parietal, occipital, and cerebellar areas, which play crucial roles in movement sequencing and coordination [59,60]. Overall, this phenomenon of attentional input disruptions can amplify the body’s natural pain-inhibitory mechanisms, potentially engaging both supraspinal systems and descending pathways that regulate the transmission of nociceptive signals at the spinal cord level [61].

Thirdly, expectation and conditioning effects may explain the immediate decrease in pain and significant improvements observed in the pressure pain threshold (PPT) within the EG. In this regard, repeated exposure to the visual illusion of pain-free movement during an MVF session may lead to the formation of positive associations between movement and reduced pain [62,63]. This can be supported with the fact that observation of a movement in a safe context can be attributed to the reduced activation of cortical regions associated with spatial localization of body parts, such as the superior parietal lobule [64], working memory and sensorimotor processing, superior frontal gyrus [65], and notably mesolimbic areas linked to emotions like the insular cortex responsible for the sense of agency [65,66]. Research on psycho-physiological interactions has illustrated the functional connections between the anterior insula and the amygdala [67], as well as the interplay between the insula and the dorsomedial frontal cortex involved in intentional movement generation [60,68,69]. This might adequately decrease the amygdala’s sensitivity in models experiencing nociceptive pain, thus diminishing the effects of catastrophizing and fear of movement commonly felt immediately after undergoing Dry Needling.

Finally, it has been demonstrated that MVF induces cortical reorganization by promoting the formation of new neuronal connections and modifying existing neuronal networks [39,70,71]. However, despite this, we rule out the possibility that the positive effects observed in our experiment are due to this mechanism. In fact, the characteristics of our design do not allow us to ascertain the neuroplastic changes that would occur, presumably, with long-term exposure to MVF.

### Limitations

Despite the promising results, this pilot study presents certain limitations that should be considered when interpreting the findings. These limitations include the relatively small sample size and the lack of long-term follow-up to assess the durability of observed MVF effects. Additionally, heterogeneity in pain severity and duration among participants could have influenced the results and may limit the generalizability of findings to a broader population of post-needling pain patients.

To advance the understanding of immediate MVF effects on post-needling pain and sensory function in lateral elbow myofascial pain patients, future research exploring different treatment protocols is warranted. This includes variations in the duration and frequency of MVF sessions, as well as the incorporation of additional outcome measures such as motor function and health-related quality of life. Furthermore, longitudinal studies with larger participant cohorts and long-term follow-up are needed to evaluate the efficacy and durability of MVF effects in managing post-needling pain across diverse clinical populations. These forthcoming studies will contribute to refining and enriching the application of MVF in clinical settings for addressing iatrogenic nociceptive pain, not only in relation to DDN but also other techniques where counterirritants, such as Dry Needling for MTrPs in the lateral elbow, play a significant role in immediate analgesic responses.

## 5. Conclusions

MVF reduced post-needling pain and influenced sensory perception with MTrPs in Lateral Elbow Pain patients. The significant pain reduction and notable trend in sensory discrimination imply positive effects of MVF. These results underscore the necessity for a more comprehensive mechanistic understanding and highlight MVF’s potential as an adjunctive intervention in managing myofascial pain.

## Figures and Tables

**Table 1:** Demographic description of the sample.

Outcomes	Group	Mean	SD	T-Student	*p*
Age (years)	EG	24.9	10.9		
	CG	24.9	9.5	−0.002	0.998
Weight (Kg)	EG	75.8	5.7		
	CG	73.4	10.6	0.629	0.538
Height (m)	EG	1.77	0.06		
	CG	1.72	0.08	1.261	0.224
BMI (Kg/m^2^)	EG	24.3	1.7		
	CG	24.7	3.5	−0.337	0.740

Note: BMI: body mass index. T-student tests for independent samples were carried out.

**Table 2:** Description of study variables.

Outcomes	Group	Mean	SD	Shapiro–Wilk W	*p*
Pre-Pain intensity	EG	1.416	1.269	0.898	0.017 **
(VAS 0-10)	CG	1.150	0.955	0.861	0.004 **
Post-Pain intensity	EG	1.064	0.934	0.857	0.002 **
(VAS 0-10)	CG	1.633	1.025	0.927	0.085
Pre-PPT	EG	1.909	0.672	0.911	0.033 *
(Kg/cm^2^)	CG	1.640	0.683	0.862	0.004 **
Post-PPT	EG	1.746	0.613	0.938	0.135
(Kg/cm^2^)	CG	1.574	0.729	0.895	0.017
Pre-TPDT	EG	9.320	5.872	0.826	<0.001 ***
(mm)	CG	13.500	6.871	0.947	0.232
Post-TPDT	EG	9.640	6.544	0.840	0.001 **
(mm)	CG	12.083	6.213	0.903	0.025 **
Pre-MHGS	EG	37.96	8.032	0.911	0.033 *
(Kg/F)	CG	34.04	7.386	0.924	0.073
Post-MHGS	EG	37.712	7.986	0.928	0.077
(Kg/F)	CG	34.867	7.609	0.944	0.204

Note: MHGS: maximum hand grip strength, PPT: pain pressure threshold, TPDT: two-point discrimination threshold, VAS: visual analogue scale. Shapiro–Wilk goodness-of-fit test was considered statistically different (*) *p* < 0.05, (**) *p* < 0.01, (***) *p* < 0.001.

**Table 3:** Intra-group differences of the experimental group.

						CI [95%]		ES
Outcomes	Test	Statistic	*p*	MD	SEM	Inf.	Sup.	Rank–Biserial
Pain intensity (VAS 0-10)	Wilcoxon W	137.00	0.047 *	0.400	0.271	0.000	Inf.	0.442
PPT (Kg/cm^2^)	Wilcoxon W	262.00	<0.001 ***	0.148	0.038	0.071	Inf.	0.747
TPDT (mm)	Wilcoxon W	121.00	0.711	−0.000	0.674	−1.500	Inf.	−0.127
MHGS (Kg/F)	Wilcoxon W	177.00	0.224	0.200	0.199	−0.150	Inf.	0.180

Note: MHGS: maximum hand grip strength, PPT: pain pressure threshold, TPDT: two-point discrimination threshold, VAS: visual analogue scale. Statistically differences were considered (*) *p* < 0.05, (***) *p* < 0.001.

**Table 4:** Intra-group differences of the control group.

						CI [95%]		ES
Outcomes	Test	Statistic	*p*	MD	SEM	Inf.	Sup.	Rank–Biserial
Pain intensity	Wilcoxon W	105.50	0.643	−0.400	0.247	−0.899	−Inf.	−0.086
(VAS 0-10)								
PPT (Kg/cm^2^)	Wilcoxon W	163.00	0.648	0.029	0.073	−Inf.	0.166	0.086
TPDT (mm)	Wilcoxon W	207.00	0.983	1.999	1.047	−Inf.	3.000	0.496
MHGS (Kg/F)	Wilcoxon W	83.00	0.081	−0.799	1.051	−Inf.	0.200	−0.343

Note: MHGS: maximum hand grip strength, PPT: pain pressure threshold, TPDT: two-point discrimination threshold, VAS: Visual Analogue Scale.

**Table 5:** Intergroup differences.

					ES
Outcomes	Test	Statistic	*p*	Rank–Biserial Correlation	Rank–Biserial Correlation
Pain intensity (VAS 0-10)	Mann–Whitney U	188.000	0.034 *	−0.373	0.165
Post-PPT (Kg/cm^2^)	Mann–Whitney U	354.000	0.862	0.180	0.165
Post-TPDT (mm)	Mann–Whitney U	212.000	0.079	−0.293	0.165
Post-MHGS (Kg/F)	Mann–Whitney U	365.000	0.905	0.217	0.165

Note: MHGS: maximum hand grip strength, PPT: pain pressure threshold, TPDT: two-point discrimination threshold, VAS: Visual Analogue Scale. Mann–Whitney U test was used for statistical differences (*) *p* < 0.05. Location parameters were obtained using the Hodges–Lehmann Estimate. Size effect was calculated using the point–biserial correlation coefficient.

## Data Availability

Data will be available on demand.

## Supplementary Materials

The following supporting information can be downloaded at: https://www.mdpi.com/article/10.3390/jcm13051490/s1, Table S1: CONSORT 2010 checklist of information to include when reporting a randomized trial.
